# Predicting the risk of iliofemoral vascular complication in complex transfemoral-TAVR using new generation transcatheter devices

**DOI:** 10.3389/fcvm.2023.1167212

**Published:** 2023-07-06

**Authors:** Ofir Koren, Vivek Patel, Yuval Tamir, Keita Koseki, Danon Kaewkes, Troy Sanders, Robert Naami, Edmund Naami, Daniel Eugene Cheng, Sharon Shalom Natanzon, Alon Shechter, Jeffrey Gornbein, Tarun Chakravarty, Mamoo Nakamura, Wen Cheng, Hasan Jilaihawi, Raj R. Makkar

**Affiliations:** ^1^ Cedars-Sinai Medical Center, Smidt Heart Institute, Los Angeles, California; ^2^ Bruce Rappaport Faculty of Medicine, Technion Israel Institute of Technology, Haifa, Israel; ^3^ Weizmann Institute of Science, Rehovot, Israel; ^4^ Department of Cardiology, The University of Tokyo Hospital, Tokyo, Japan; ^5^ Queen Sirikit Heart Center of the Northeast, Department of Medicine, Faculty of Medicine, Khon Kaen University, Khon Kaen, Thailand; ^6^ David Geffen School of Medicine, University of California (UCLA), Los Angeles, California; ^7^ Internal Medicine, University Hospitals Cleveland Medical Center, Case Western Reserve, University School of Medicine, Cleveland, United States; ^8^ School of Medicine, University of Illinois, Chicago, IL, United States; ^9^ Faculty of Medicine, Tel Aviv University, Tel Aviv, Israel; ^10^ Department of Cardiology, Rabin Medical Center, Petach Tikva, Israel

**Keywords:** TAVR, iliofemoral vascular complications, tortuosity, risk model, validation & verification component, calcification, aortic stenosis, crossability

## Abstract

**Objective:**

Design a predictive risk model for minimizing iliofemoral vascular complications (IVC) in a contemporary era of transfemoral-transcatheter aortic valve replacement (TF-TAVR).

**Background:**

IVC remains a common complication of TF-TAVR despite the technological improvement in the new-generation transcatheter systems (NGTS) and enclosed poor outcomes and quality of life. Currently, there is no accepted tool to assess the IVC risk for calcified and tortuous vessels.

**Methods:**

We reconstructed CT images of 516 propensity-matched TF-TAVR patients using the NGTS to design a predictive anatomical model for IVC and validated it on a new cohort of 609 patients. Age, sex, peripheral artery disease, valve size, and type were used to balance the matched cohort.

**Results:**

IVC occurred in 214 (7.2%) patients. Sheath size (*p* = 0.02), the sum of angles (SOA) (*p* < .0001), number of curves (NOC) (*p* < .0001), minimal lumen diameter (MLD) (*p* < .001), and sheath-to-femoral artery diameter ratio (SFAR) (*p* = 0.012) were significant predictors for IVC. An indexed risk score (CSI) consisting of multiplying the SOA and NOC divided by the MLD showed 84.3% sensitivity and 96.8% specificity, when set to >100, in predicting IVC (C-stat 0.936, 95% CI 0.911–0.959, *p* < 0.001). Adding SFAR > 1.00 in a tree model increased the overall accuracy to 97.7%. In the validation cohort, the model predicted 89.5% of the IVC cases with an overall 89.5% sensitivity, 98.9% specificity, and 94.2% accuracy (C-stat 0.842, 95% CI 0.904–0.980, *p* < .0001).

**Conclusion:**

Our CT-based validated-model is the most accurate and easy-to-use tool assessing IVC risk and should be used for calcified and tortuous vessels in preprocedural planning.

## Introduction

Transcatheter aortic valve replacement (TAVR) has become the standard of care, and a long-established therapeutic approach for severe aortic stenosis (AS) patients at high- and intermediate-risk profiles. Recently it has extended to young patients of low risk (1–4).

The transfemoral-TAVR (TF-TAVR) approach is, without doubt, the most widely used access site due to its relatively low rates of major vascular injury, it's higher rate of five-year survival and the long operator experience established with coronary interventions (5, 6).

The PARTNER studies reported iliofemoral vascular complication (IVC) in almost a quarter of TAVR patients, following the standardized endpoint definition by the Valve Academic Research Consortium (VARC), with an equal distribution among major and minor complications (6–8). The routine uses of computed tomography (CT) for pre-procedural planning and the technological improvement in the transcatheter device, the sheath profile and the vascular closure device has substantially reduced the rate of major IVC to 4%. Yet, the tremendous reduction has modestly aid in solving a daily-life practical dilemma for calcified, narrow, and tortuous vessels which is seen in up to 40% of TAVR patients (9–11).

IVC is strongly associated with increased mortality, bleeding complications, and hospital readmissions. It has a higher rate of renal impairment, access site infections, prolonged hospitalizations, and requires more blood transfusions (12, 13).

Among known anatomical predictors for IVC, such as minimal lumen diameter, extensive iliofemoral calcification, low sheath-to-femoral artery diameter ratio (SFAR) and multiple large-bore sheath entries, tortuosity is the only parameter still routinely being reviewed by visual assessment (14–17).

Despite the comprehensive progression in research and operator experience, there is no acceptable tool to guide operators to safely use the transfemoral arteries in cases of severe calcification or tortuosity.

Our study aims to develop a risk score to quantify tortuosity and calcification of the iliofemoral arteries in a contemporary era using new generation transcatheter systems (NGTS).

## Methods

### Study design and patients' selection

We conducted a single-center retrospective study of 3,119 consecutive patients with severe symptomatic AS who underwent TAVR between 2016 and December 2021 using NGTS. All patients underwent pre-procedural high-resolution CT and were followed by a dedicated TAVR team at 30 days, 6 months, and yearly after the procedure. A consensus decision from the multidisciplinary cardiac team determined the use of TAVR for all patients and recommended the preferred access site and the type of valve choice. The access site was determined by the operators’ experience. The size of the transfemoral sheath was determined by the manufacturer's recommendation based on the type and size of the bioprosthetic valve.

All TAVR patients over 18 years old who had major and minor vascular complications, per the VARC-3 definition (18), were included in our study. We excluded patients with significant vascular complications due to closure device failure and patients with access-related non-vascular complications (infection, local hematoma, hernia), or any vascular complications above the abdominal aorta. Device closure failure due to calcified or tortuous vessels was included. Intra-operative angiography adjudicated the source of a hematoma (i.e., access-related vs. not). Additional exclusion criteria were missing patient information, poor CT images, incomplete follow-up, old generation or experimental THVs, and if planned alternative access (other than transfemoral) was performed.

**Figure 1:**
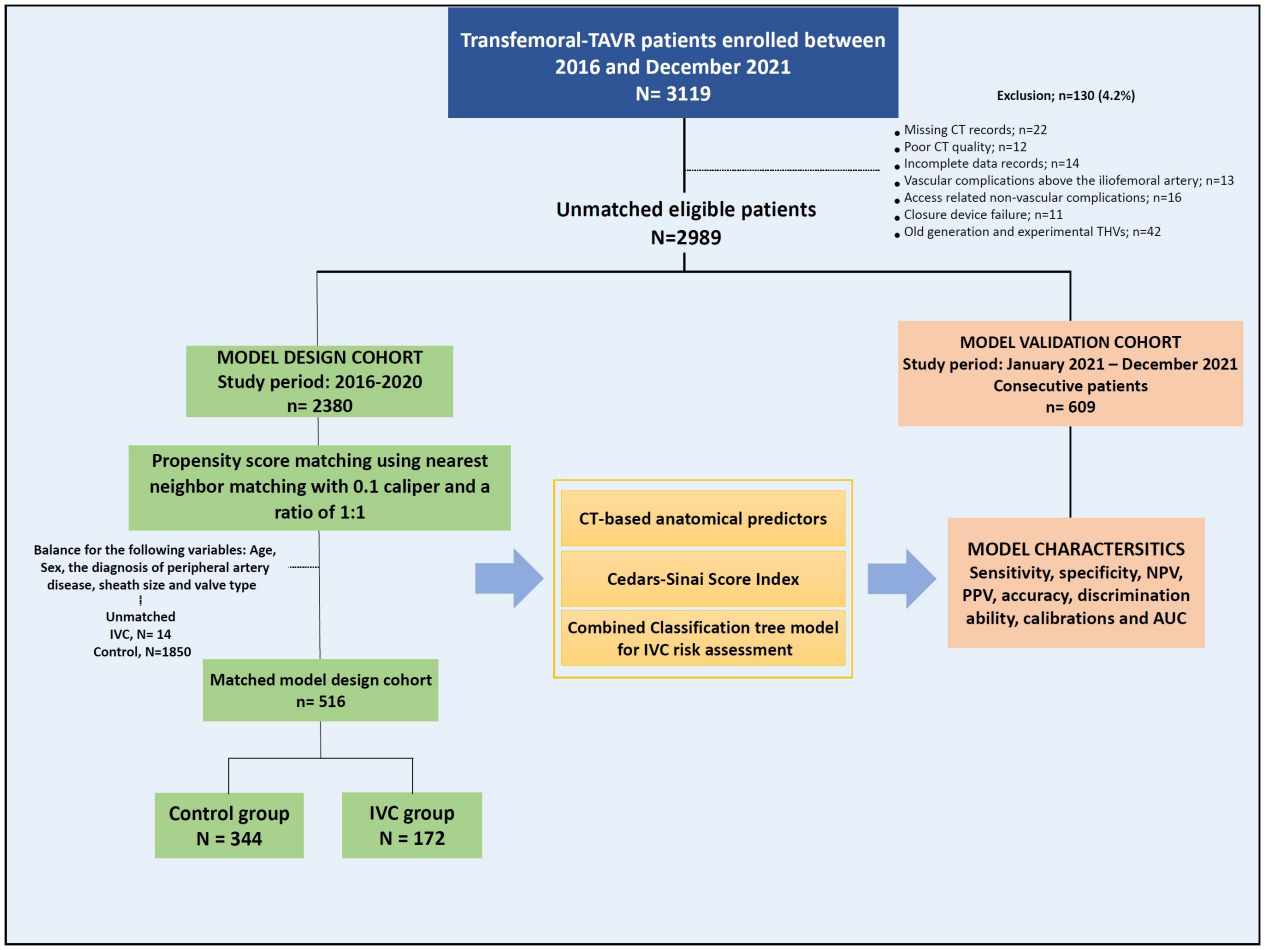
Study design.

We used the data of 2,380 patients enrolled between 2016 and 2020 to design the predictive model and validated it on 609 consecutive TAVR patients from January to December 2021 (Figure 1).

The study was approved by institutional review board (IRB), which also waived the requirement to obtain informed consent due to the study's retrospective nature.

### Definitions

IVC is defined as any major and minor vascular complications involving the iliofemoral artery as enumerated by Valve Academic Research Consortium 3 (7). This included perforation, rupture, dissection, stenosis, ischemia, thrombosis, arteriovenous fistula, pseudoaneurysm, retroperitoneal hematoma, and infection. Furthermore, we included cases of distal embolization from a vascular source, unplanned endovascular or surgical intervention. We excluded patients with IVC due to closure device failure (when not related to calcific or tortuous anatomy) or access-related non-vascular complications to ensure that our model relies mainly on the patient's anatomy and plaque characteristics (Table 1). Calcified plaques were classified into four categories based on their occupying portion of the lumen and the perimeter (Table 2B).

**Table 1:** Distribution of Iliofemoral Vascular Complications.

Types of Iliofemoral Vascular Complications	Unmatched population (*N* = 214)		Matched population (*N* = 172)	
Dissection	77	36.0%	62	36.0%
Perforation	53	24.8%	43	25.0%
Pseudoaneurysm	42	19.6%	34	19.8%
Stenosis, Thrombosis, Distal embolization or Lower Extremity Ischemia	36	16.8%	28	16.3%
AV fistula	6	2.8%	5	2.9%
	TOTAL 214	100.0	TOTAL 172	100.0

**Table 2:** Univariate and multivariate analysis of CT based anatomical predictors for IVC in matched population.

Variable	Control Group	IVC group	Univariate			Multivariable		
	*N* = 344 (%)	*N* = 172 (%)	OR	95% CI	*p*-Value	OR	95% CI	*p*-Value
Sum of all angles	110 (57)	165 (75)	1.42	0.842–1.944	<.0,001	1.28	1.024–1.664	<.0,001
Single maximum angle	68 (11)	65 (11)	0.98	0.767–1.022	.342			
Minimal lumen area, mm^2^	52.4 (6)	36.4 (6)	−1.38	0.998–1.648	.001	−1.18	0.987–1.241	.004
Minimal lumen perimeter, mm	28.9 (4)	20.2 (3)	−1.44	0.774–1.648	.001	−1.16	0.898–1.488	.002
Minimal lumen diameter, mm	6.8 (2)	5.6 (1)	−2.24	0.987–3.214	<.0,001	−2.08	1.644–4.428	<.0,001
Number of curves, mm	3.4 (1)	5.6 (2)	2.79	0.994–3.398	<.0,001	2.34	1.064–3.342	<.0,001
Mean of all lumen diameters, mm	20.4 (3)	28.4 (4)	1.42	0.789–1.842	.004	1.08	0.889–1.136	.045
Mean distance between curves, mm	61.9 (13)	42.4 (11)	1.28	0.648–1.441	.002	1.11		.052
Calcification Category (CAG)≥ III*	75 (21.8)	58 (33.7)	2.11	0.978–3.644	<.0,001	1.98	1.142–4.582	<.0,001
IFT^β^	20.4 (5)	23.4 (6)	1.12	0.784–1.268	.001	1.07	0.787–1.178	.024
Sheath size > 14F, n (%)	113 (32.8)	56 (32.6)	1.01	0.664–1.142	.244			
SFAR	0.86 (0.2)	1.09 (0.2)	1.46	0.977–1.684	<.0,001	1.29	1.021–2.412	.001

IFT, iliofemoral tortuosity; SFAR, the ratio of the sheath outer diameter to femoral artery minimal lumen diameter.

* Calcification category per Suppementary Table S2b.

^β^ Calculated using the formula: $\mathrm{IFT}=\left(\frac{\mathrm{true}\;\mathrm{vessel}\;\mathrm{length}\;}{\mathrm{ideal}\;\mathrm{vessel}\;\mathrm{length}\;}-1\right)x100$^24.^

Collinearity Collison was observed among the following variables: minimal lumen diameter, minimal lumen perimeter and minimal lumen area.

**Table 2B:** Calcification Category

Category	Proportional of the Perimeter (%)	Proportional of the Lumen (%)
I	<50%	<50%
II	≥50%	<50%
III	<50%	≥50%
IV	≥50%	≥50%

### Multidetector computed tomography

The images were analyzed offline at the Cedars-Sinai Heart Institute Core Laboratory using the 3mensio Valves software version 9.0 (3mensio Medical Imaging) by a designated and experienced team.

### CT-Based measurments

The length of the iliofemoral artery was defined fluoroscopically, from the puncture site, above the superficial and deep femoral bifurcation and below the inferior epigastric artery, up to the abdominal aortic and common iliac artery bifurcation. We analyzed iliofemoral artery injuries at the site of the large bore sheath. Alignment of the central line was done manually for each patient and the curve's angle was measured automatically using the tortuosity mode at the vessel's volume rendering view. We determined the curve' maximum angle by measuring the highest angle at the peak of the curve and used the ruler tool to measure the minimal lumen diameter in the perpendicular plane views.

To assess the effect of plaque on a curve's angle (POC), we measured the angle with and without alignment of the central lumen line for plaques located either on the tip of the curve's vessel, at the bottom, or both (circumferential plaques) using the snake view (Supplementary Figure S1).

### Valve types, vascular closure devices and sheaths

Two types of NGTS were used in our cohort: intra-annular balloon-expandable SAPIEN-3 or Sapien-Ultra valve using an e-Sheath delivery system [Edwards Lifesciences, Irvine, California, USA) and the supra-annular self-expanding Evolut-R, or Evolut-PRO valve along using the EnVeo R InLine™ sheath [Medtronic, Minneapolis, MN, USA]. The vascular closure device used in all the cases was the Perclose ProGlide system [Abbott Vascular, Santa Clara, CA, USA].

### Statistical analysis

Continuous variables were compared between vascular complication vs. no complication groups using the 2-tailed student's t-test or Mann Whitney U test and presented as mean ± standard deviation or median and interquartile range, respectively. Categorical variables were expressed as frequencies and percentages and compared using the chi-square or Fisher exact test.

The propensity score-based matching (PSM), using logistic regression with the nearest-neighbor creating two study groups with and without IVC complications. Variables with a *p*-value higher than.01 in the multivariable regression analysis and patients with deviated threshold scores (caliper) of >0.10 were excluded from PSM. Due to the relatively low incidence of IVC and to empower the effect of the model, we chose the two-to-one matching ratio (19).

Multivariable logistic regression was performed using the minimum Akaike Information criteria (minimum AIC) for variable selection using all CT variables with *p* < 0.05 in the univariate analysis as candidates. We used the Pearson's linear correlation test to assess the correlation strength and polarity of the MLD and the probability of IVC and the Spearman's correlation test for the number of curves and the sum of the curve's angle.

The CSI formula was constructed using the integration of statistically significant variables found by using the logistic regression, the correlation polarity and through their contributions to predicting IVC.

We assessed accuracy by computing receiver operating curves (ROC) and reporting the ROC area (concordance statistic C) and the sensitivity and specificity at maximum accuracy. In addition to the logistic models, we used a binary recursive partition (classification tree) model to determine the optimal thresholds for our final CSI and SFAR predictors of vascular complications. Thus, the predictor variables were found using a logistic search and the thresholds for the final variables found were obtained *via* a classification tree.

The minimum sample size needed to validate the model is 450 patients, assuming at least an incidence rate of 6.5% IVC, with a 97.7% sensitivity, 96.8% specificity, and a 95% confidence bound width of 8.9%, found in the model design cohort.

Two-sided *p* values were considered significant if they were less than 0.05. All statistical analyses were performed using JMP version 15.2.0 (SAS Institute) and SPSS statistical package, version 24.0 (SPSS Inc., Chicago, IL, USA).

## Results

### Study population

Overall, 3,119 patients underwent TAVR using the transfemoral approach between 2016 and 2021 at Cedars-Sinai medical center. We excluded 130 (4.2%) patients for the following reasons: 42 patients for using old generation transcatheter device system, 36 patients for incomplete electronic data records and missing imaging records, and 12 patients due to poor imaging quality. We also excluded 13 patients with vascular complications above the iliofemoral segment, 16 patients with access-related non-vascular complications and 11 with closure device failure.

Among the 2,989 eligible patients, enclosing the model design and the model validation cohorts, 214 (7.2%) patients had IVC with no significant difference between the cohorts (7.8 vs. 7.2%, respectively, *p* = .465).

In the unmatched population, IVC was more common in old patients (82.7 vs. 80.9%, *p* < .0001), females (49.2 vs. 38.3%, *p* = .001), and in patients with peripheral artery disease (23.7 vs. 13.4%, *P* < .0001). The use of sheath size of >14F and the use of self-expanding transcatheter valve was more common in the IVC group (28.2 vs. 19.2%, and 22.9 vs. 17.8%, respectively, *p* = .001, *p* = .046). This group also exhibited a higher median Society of Thoracic Surgeons (STS) risk score (5.0 vs. 3.5, *p* < .0001) and a lower Kansas City Cardiomyopathy Questionnaire (KCCQ) score (51.3 vs. 56.2, *p* = .046). No statistically significant differences were noted in the aortic valve area, left ventricular ejection fraction, and the total calcium score of the aortic valve or the coronary arteries between the groups (Supplementary Table S1).

### Pre-Procedure characteristics and outcomes in a matched cohort

**Figure 2:**
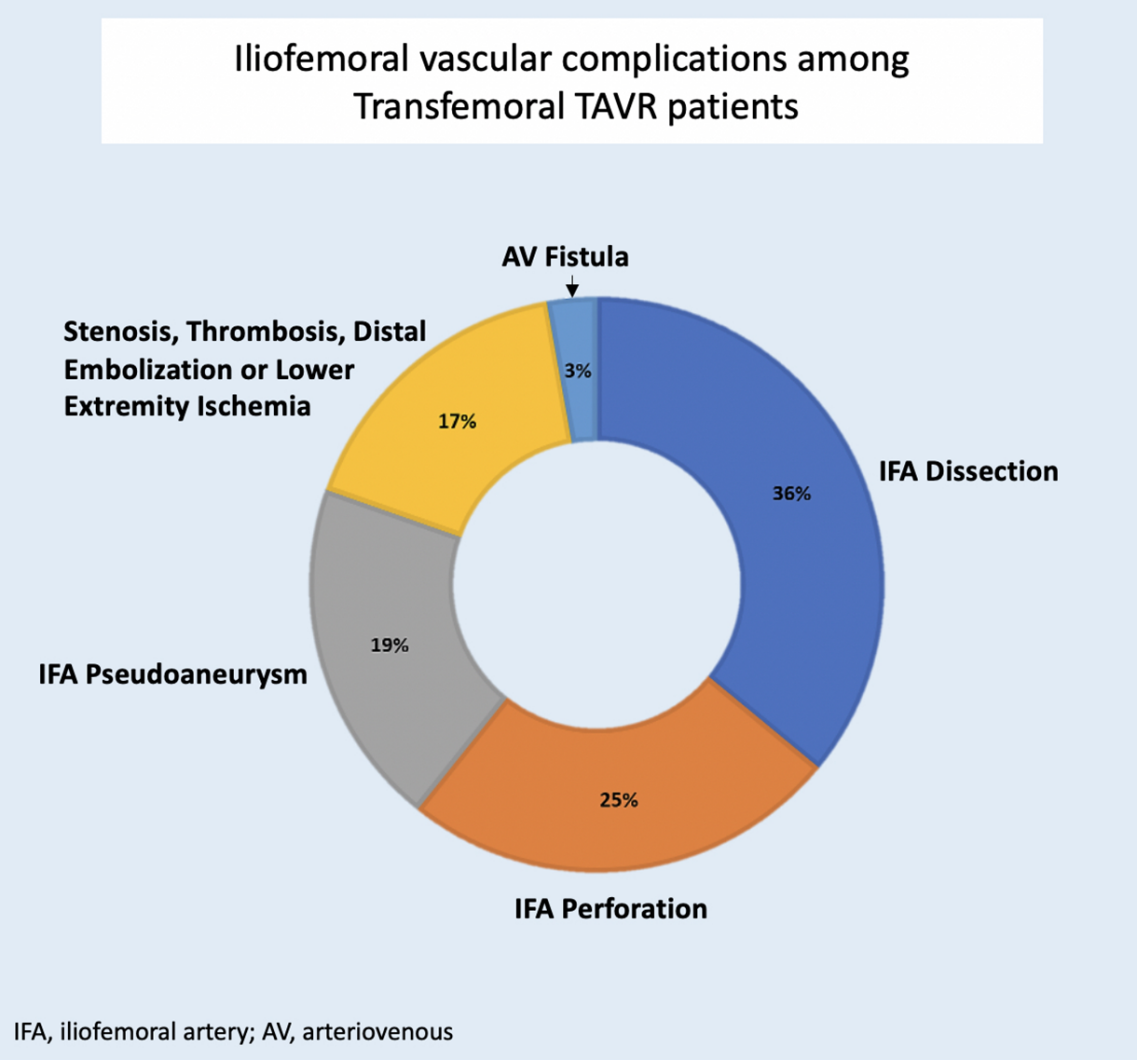
Distribution of Iliofemoral Vascular Complications: Matched Cohort.

The matched model design cohort was balanced for the following variables: age, sex, sheath size, and transcatheter-valve type (Supplementary Figure S2) adopted from the multivariable regression analysis and includes a total of 516 patients; 172 patients in the IVC group were compared to 344 patients with no complications, which comprised of the Control group. An overall of 14 IVC patients and 1,850 control patients were unmatched and excluded from the analysis (Figure 1). Of the 172 matched patients, iliofemoral complications were distributed as followed: 62 dissection injury (36%), 43 perforation (25%), 34 pseudoaneurysm (19.8%), 28 stenosis, thrombosis, distal embolization or lower extremity ischemia (16.3%), and 5 AV Fistula (2.9%) (Table 1, Figure 2).

IVC patients had more use of contrast volume (90 vs. 65 ml, *p* < .0001), longer fluoroscopy time (18.3 vs. 12.7, min *p* < .0001), more extended post-anesthesia care unit (PACU) and hospital stay (6.4 vs. 4.6, median hours, *p* = .008 and 3.3 vs. 2.0, days, *p* < .0001, respectively) and longer procedure duration (01:50 vs. 01:18, mean hours hh: mm, *p* < .0001). Furthermore, IVC patients exhibit higher rates of major and minor procedural-related bleeding (8.1 vs. 4.6%, *p* < .0001), in-hospital stroke (4.7 vs. 1.7%, *p* = .001), and 30-days hospital readmission for any cause (7.6 vs. 2.3%, *p* = .008). A statistical trend was noticed in 30-day mortality rate toward the IVC group (2.3 vs. 0.6%, *p* = .059) (Table 3).

**Table 3:** Patients’ characteristics, procedural data, and outcome in matched populations.

	Total study population *N* = 516	Matched IVC Group* *N* = 172	Matched Control Group* *N* = 344	*p*-Value
Age (years), median ± SD (IQR)	82.0 ± 9.5 (13)	82.5 ± 8.81 (15)	81.0 ± 9.8 (13)	.892
Female Sex, *n* (%)	281 (52.5)	101 (52.7)	180 (52.3)	.889
Smoking, *n* (%)	15 (2.9)	5 (2.9)	10 (2.9)	1.00
BMI, *n* (%)	28.1 ± 24.1 (7)	26.83 ± 6.5 (8)	28.71 ± 28.1 (7)	.993
CKD, *n* (%)	102 (19.8)	40 (23.3)	62 (18.0)	.162
Dialysis, *n* (%)	38 (7.4)	15 (8.8)	23 (6.7)	.377
COPD, *n* (%)	110 (21.5)	43 (25.1)	67 (19.7)	.172
Hyperlipidemia, *n* (%)	287 (55.6)	99 (57.6)	188 (54.7)	.573
Diabetes Mellitus, *n* (%)	166 (32.3)	55 (32.2)	111 (32.4)	1.00
Coronary artery disease, *n* (%)	230 (44.6)	83 (48.3)	147 (42.7)	.260
Peripheral artery disease, *n* (%)	73 (14.1)	24 (13.9)	49 (14.2)	.897
CVA/TIA, *n* (%)	65 (12.6)	22 (12.8)	43 (12.5)	.514
Myocardial Infarction, *n* (%)	62 (12.1)	21 (12.3)	41 (12.0)	1.00
CABG surgery, *n* (%)	83 (16.1)	27 (15.6)	56 (16.2)	.646
Porcelain Aorta, *n* (%)	13 (2.5)	4 (2.3)	9 (2.6)	1.00
STS risk score, median ± SD (IQR)	5.44 ± 6.7 (7)	5.62 ± 8.2 (8)	5.30 ± 5.7 (6)	.067
NYHA Class≥ III, *n* (%)	412 (79.8)	137 (79.6)	275 (79.9)	.846
Five minutes’ walk test (m), median ± SD (IQR)	16.0 ± 6.9 (6)	15.89 ± 6.2 (5)	16.77 ± 7.1 (6)	.541
KCCQ Score, median ± SD (IQR)	54.1 ± 24.4 (40)	51.06 ± 22.7 (36)	54.62 ± 25.1 (39)	.180
Balloon-expandable THV, *n* (%)	381 (73.9)	127 (73.4)	254 (73.9)	.898
Old Generation THV, *n* (%)	102 (19.7)	34 (19.7)	68 (19.7)	.997
Large THV^¥^, *n* (%)	164 (31.8)	52 (30.2)	112 (32.6)	.617
Aortic valve area (mm), median ± SD (IQR)	0.7 ± 0.2 (0)	0.70 ± 0.2 (0)	0.70 ± 0.19 (0)	.956
Coronaries Ca Score (HU), median ± SD (IQR)	932.0 ± 1,720.1 (1,695)	1,433.6 ± 1,679.9 (1,916)	911.0 ± 1,741.8 (1,666)	.740
AV Ca score (HU), median ± SD (IQR)	2,446.0 ± 2,050.4 (2,379)	1,982.5 ± 2,000.3 (1,963)	2,607.0 ± 2,065.0 (2,357)	.100
LVEF (%)^,^ median ± SD (IQR)	64.0 ± 13.2 (14)	62.0 ± 12.1 (14)	64.0 ± 13.7 (14)	.241
Contrast volume (ml), median ± SD (IQR)	70.0 ± 50.8 (50)	90.0 ± 51.2 (50)	65.0 ± 48.5 (35)	<.0,001
Fluoroscopy time (min), median ± SD (IQR)	14.1 ± 10.5 (11)	18.3 ± 10.6 (13)	12.7 ± 9.9 (9)	<.0,001
PACU time (hours), median ± SD (IQR)	5.5 ± 1.8 (2.1)	6.4 ± 1.4 (2.1)	4.6 ± 1.6 (2.3)	.008
Length of hospitalization (days), median ± SD (IQR)	2.0 ± 2.6 (1)	3.3 ± 3.1 (2)	2.0 ± 2.3 (2)	<.0,001
Procedure Duration (hh:mm), median ± SD (IQR)	1:26 ± 0:58 (0:55)	1:50 ± 0:53 (1:17)	1:18 ± 0:57 (0:50)	<.0,001
Procedural related major and minor Bleeding, *n* (%)	30 (5.8)	14 (8.1)	16 (4.6)	<.0,001
Acute kidney injury, *n* (%)	46 (8.9)	17 (9.9)	29 (8.4)	.624
In-hospital mortality, *n* (%)	8 (1.6)	1 (0.6)	7 (2.0)	.279
30-day mortality, *n* (%)	6 (1.2)	4 (2.3)	2 (0.6)	.059
1-year mortality, *n* (%)	36 (7.0)	14 (8.1)	22 (6.4)	.468
30-day hospital readmission, *n* (%)	21 (4.1)	13 (7.6)	8 (2.3)	.008
1-y hospital readmission, *n* (%)	20 (3.9)	10 (5.8)	13 (3.8)	1.00
In hospital stroke, *n* (%)	14 (2.7)	8 (4.7)	6 (1.7)	.001

BMI, body mass index; CKD, chronic kidney disease stage≥ III; COPD, chronic obstructive lung disease; CVA/TIA, cerebrovascular accident/Transient ischemic attack; CABG, coronary artery bypass graft; KCCQ, Kansas city cardiomyopathy questionnaire; THV, transcatheter heart valve; Ca, calcium score; HU, Hounsfield units; LVEF, left ventricular ejection fraction; PACU, post-anesthesia care unit.

^¥^ Large THV referred to 29 mm Sapien valve or 34 mm Evolute valve.

* Propensity matching was performed for the variables: age, sex, peripheral artery disease, sheath size and valve type using the nearest neighbor matching with 0.1 caliper and a ratio of 2:1. Overall balance test (Hansen & Bowers, 2,010) *p* = .982.

### CT-Based anatomical predictors

Our database shows the ideal total iliofemoral stretched length is 205 ± 13 mm, with 3 curves created while entering and exiting the great pelvic. The iliofemoral vessels of IVC patients were characterized by significantly more curves (5.6 vs. 3.4, OR 2.34, 95% CI 1.064–3.342, *P* < .0001), with acute angles (165 vs. 110, for the sum of angles, OR 1.28, 95% CI 1.024–1.664, *P* < .0001), and smaller minimal lumen diameter (MLD) (5.6 vs., 6.8, OR −2.08, 95% CI 1.644–4.428, *p* < .0001) than the control group. The maximal angle, which reflects the highest single maximum angle across the iliofemoral artery, was not significantly different among the study groups (*p* = 0.98). The minimal lumen area and perimeter show a relatively same result as the MLD (OR −1.18, OR −2.08, *P* = .004, and *P* = 0.02, respectively) with a slightly lower odds ratio. Furthermore, the IVC group had more severe vascular calcification, with a calcified plaque occupation of at least half the lumen and/or the perimeter in almost a third of the IVC patients (33.7 vs. 21.8%, for plaque category ≥ III, OR 1.98, 95% CI 1.142–4.582, *P* < .0001). The ratio of the sheath outer diameter and the MLD (SFAR) was significant among the two groups with an OR of 1.29 (95% CI 1.021–2.412, *P* = .001), while sheath size of > 14F did not differ (*p* = .244). The iliofemoral tortuosity (IFT) index assessed by the ratio between the true vessel length and the ideal vessel length (23) show only a modest effect (OR 1.07, 95% CI 0.787–1.178, *p* = .024) (Table 2).

### Cedars-Sinai Index score

We used strong anatomical predictors from the multivariable analysis and assessed the correlation and the polarity with the probability of IVC. We chose the MLD over the minimal lumen area and the perimeter due to its strength and the simplicity of acquiring its measurement. The MLD shows a negative linear behavior with a strong correlation (*r* = −0.69). In contrast, the number of curves and the sum of the curve's angle show a positive non-linear correlation (*τ* = 0.71, *τ *= 0.56, respectively, *p* < .0001 for both). The CSI score, utilizing the polarity of the above predictors, assist us in formulating a quotient consisting of multiplying the sum of angles and the number of curves divided by the minimal lumen diameter and showed the strongest correlation (*τ* = 0.76, *p* < .0001) (Supplementary Figure S3). The CSI score was the most accurate and straightforward to use mathematically based formula among eight different models created by Regression equations. Furthermore, the CSI score had the highest sensitivity, specificity, and accuracy in our cohort. Following a ROC analysis, a CSI score of over 100 resulted in an 84.3% sensitivity, 96.8% specificity, and 90.6% accuracy in predicting IVC (C-stat 0.936, 95% CI 0.911–0.959, *p* < .0001) (Figure 2).

SFAR> 1.05, as reported in prior studies (12), had lower sensitivity and did not add value to the specificity of the CSI. Lowering the SFAR threshold to> 1.00 increases the sensitivity of the model and contribute to its accuracy (79.0%, 99.7%, 89.3%, sensitivity, specificity, and accuracy, respectively, C-stat 0.878, 95% CI 0.846–0.910, *p* < .0001) (Figure 2).

**Figure 3:**
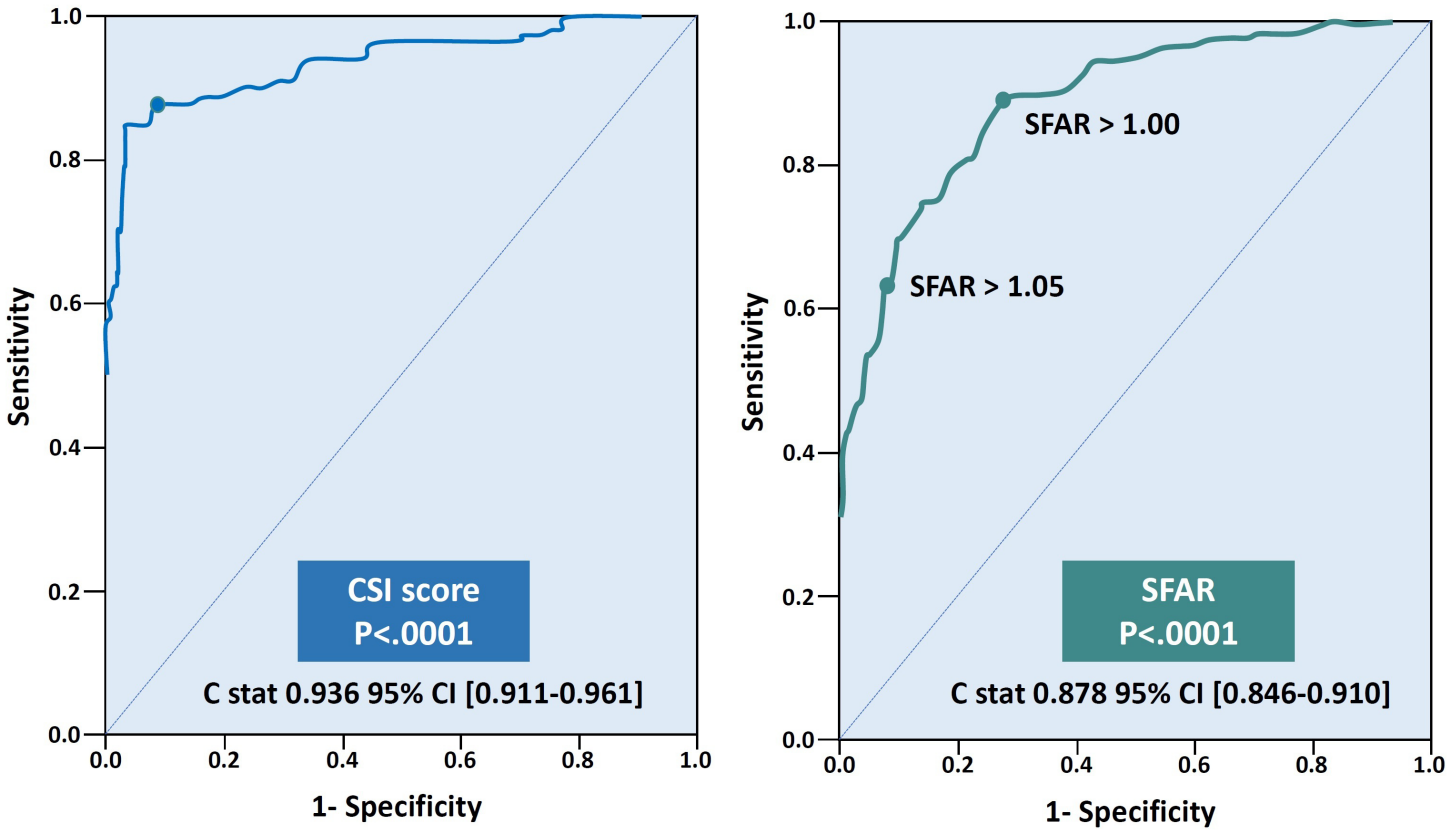
Receiver operating characteristic (ROC) curve of the CSI and the SFAR.

The distribution of the CSI and the SFAR scores in the IVC group reveal four distinct anatomical-based causes that led to the development of IVC (t (18.43), t(20.0), for SFAR and CSI, respectively, *p* < .0001 for both). IVC most commonly happens in patients with SFAR≤ 1.00 and CSI score> 100 (QIV), indicating that the main reason for the IVC was the use of a large-diameter sheath on a proportionally narrow vessel in a tortuous vessel followed by patients with SFAR> 1.00 and CSI> 100 (QIII) indicating that tortuosity was the main reason over apparently large diameter vessels. The smallest IVC group demonstrates patients where IVC developed in small and borderline-sized-diameter (QII). The highest density of event-free is seen in patients with SFAR≤ 1.00 and CSI≤ 100 (Figure 3).

### Desiging A classification tree model

**Figure 4:**
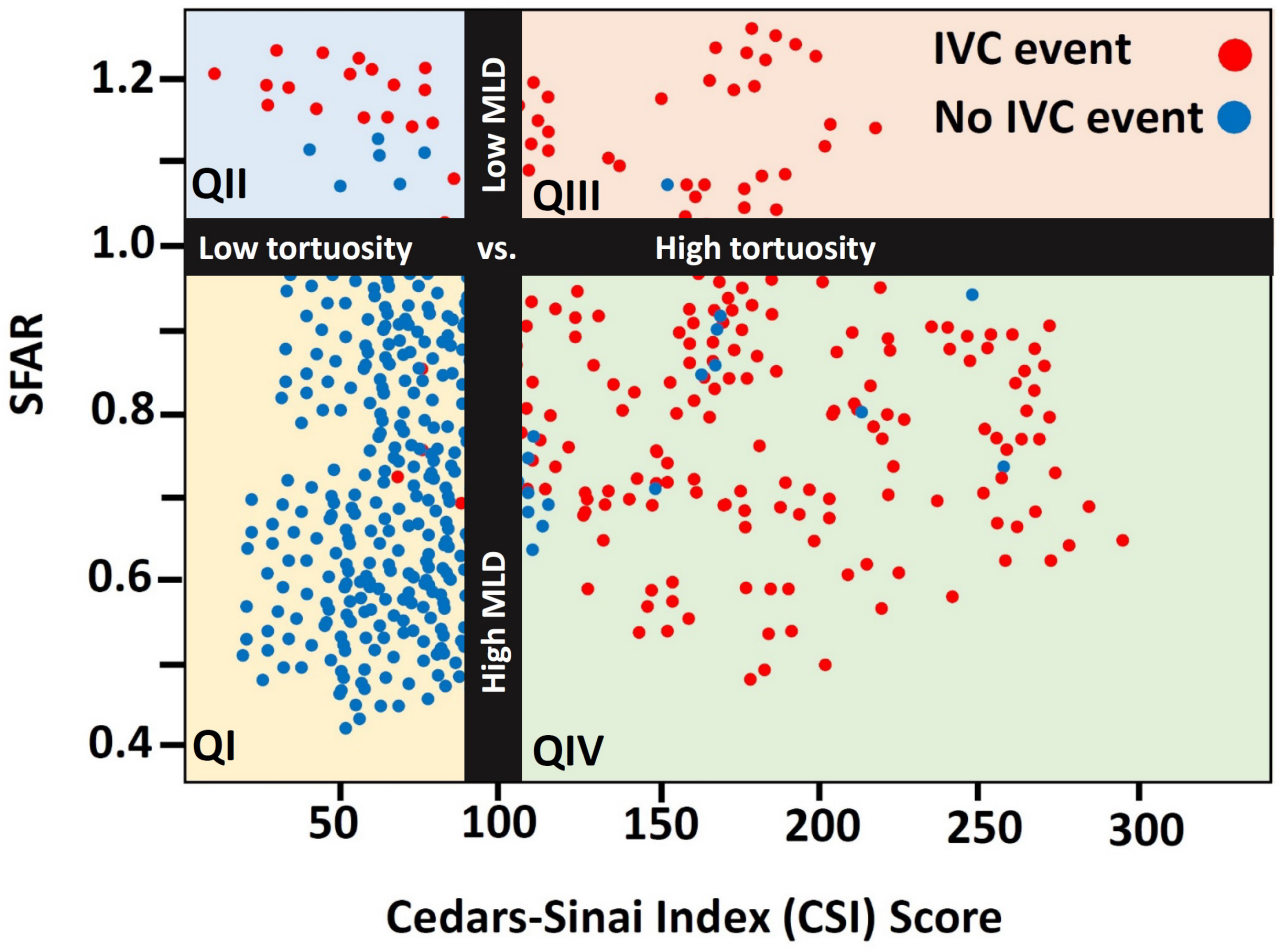
Distribution of the IVC event stratified by SFAR and CSI score.

The final designed model consists of two conditional steps, where SFAR >1.00 placed first and CSI score> 100 placed second. SFAR> 1.00 was seen in 68 patients, with 66 (97.1%) of them had IVC (B 10.98, SE (B) 1.27, *P* < .0001). CSI score higher than 100 predicts IVC in 153 patients (B 11.0, SE (B) 1.43, *P* < .0001) where 146 (95.4%) patients had vascular complication and 44 patients had also SFAR> 1.00. The model failed to predict IVC in 4 (2.3%, PPV 97.7%) patients and wrongfully predicted IVC in 9 (3.1%, NPV 96.9%) patients. The model's overall sensitivity, specificity, and accuracy were 97.7%, 96.8%, and 97.2%, C-stat 0.975. 95% CI 0.959–0.988 (Figure 4, Supplementary Table S3).

**Figure 5:**
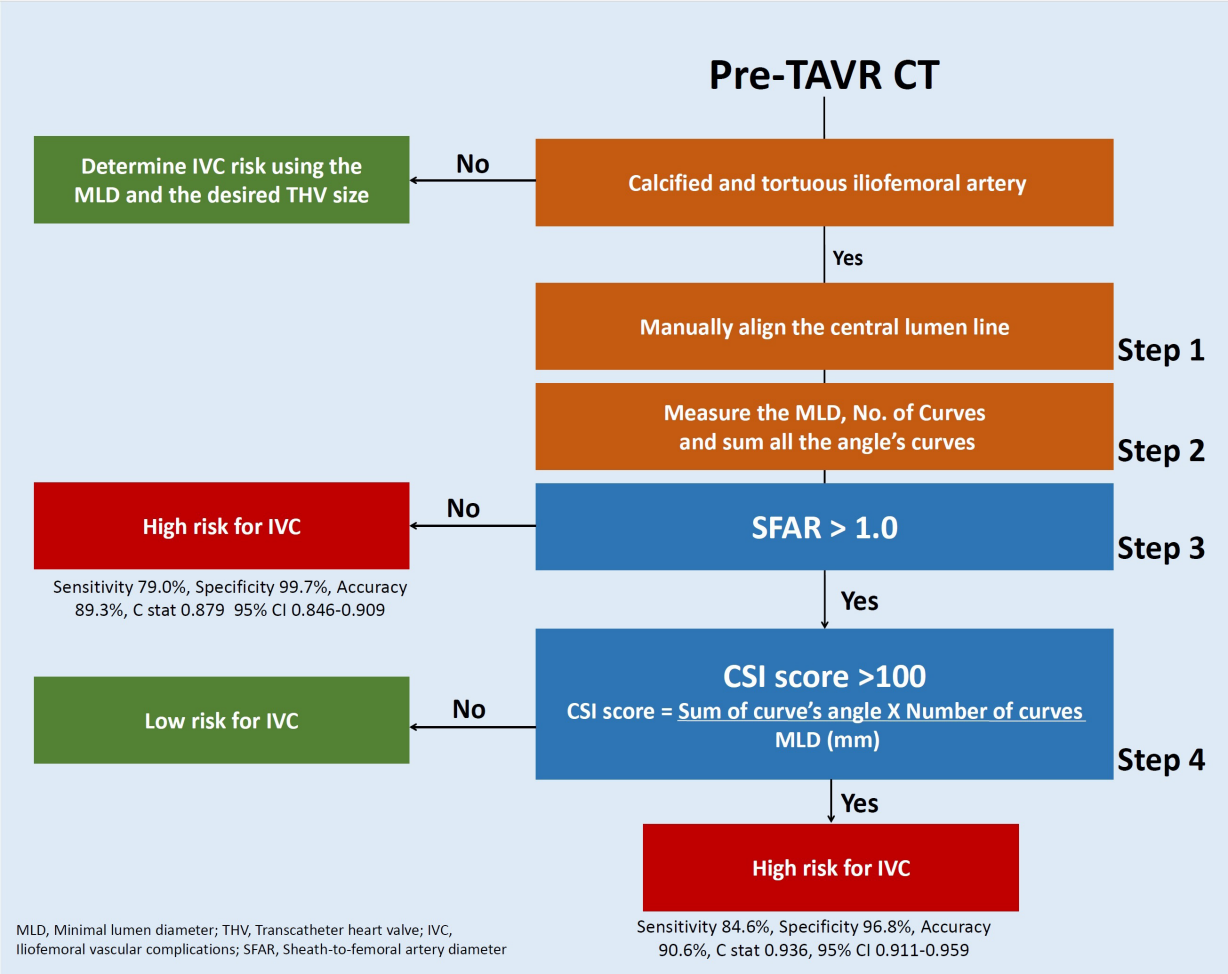
Approach to IVC risk assessment of calcified and tortuous iliofemoral artery using Cedars-Sinai Index score and the prediction model.

The final configuration of the CSI score outweighs any alternative formulas, mathematical equations, and prior assessment techniques, like the iliofemoral tortuosity score, SFAR >1.05, manufacturer minimal lumen diameter, and the current practice of using eyeballing assessment in each criterion of the overall sensitivity, specificity, accuracy, NPV, PPV, and F1 score (Figure 5).

### Effect of the Poc on curve's angle

**Figure 6:**
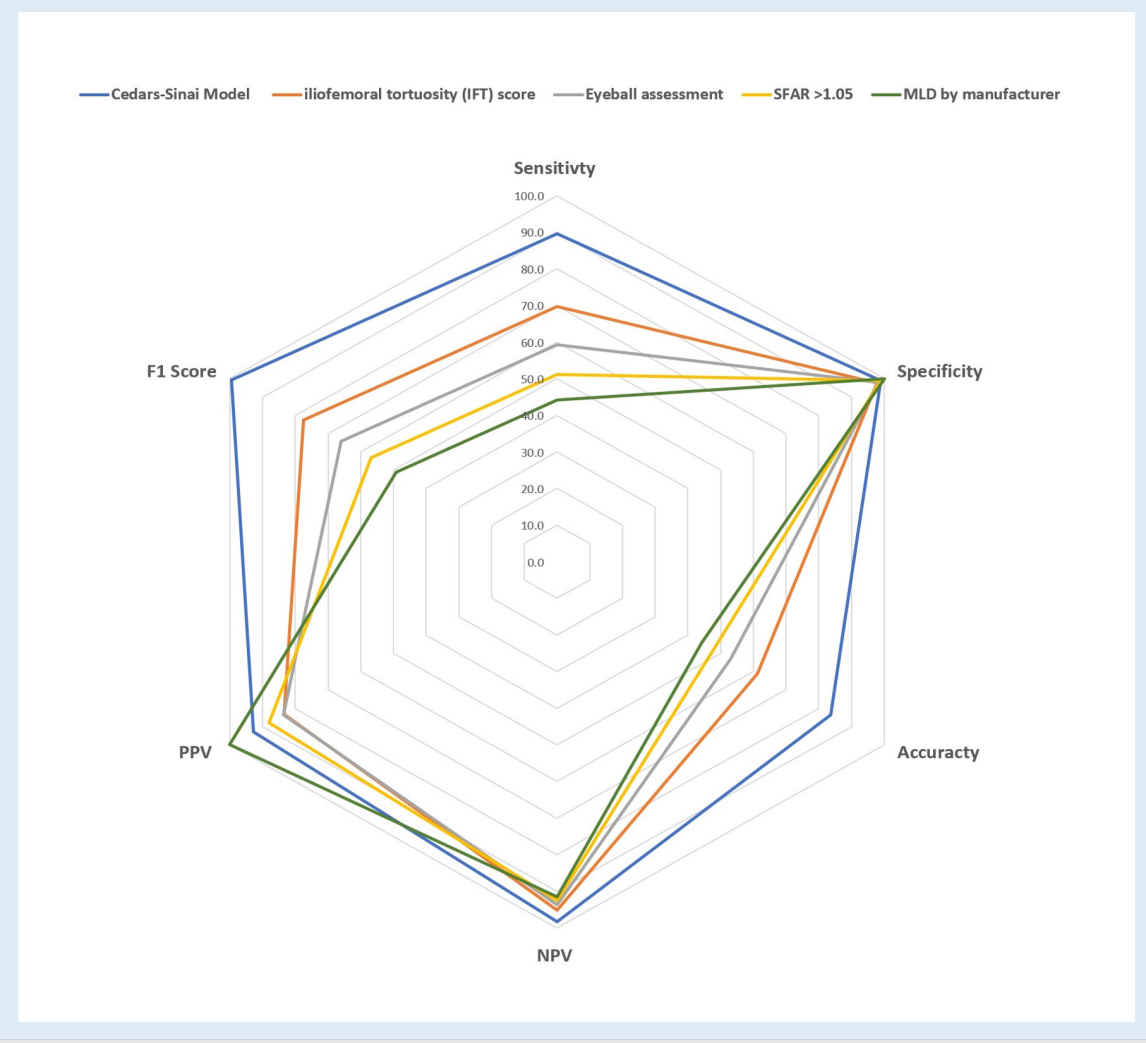
Cedars-Sinai proposed predicitive model vs. Current used methods.

**Figure 7:**
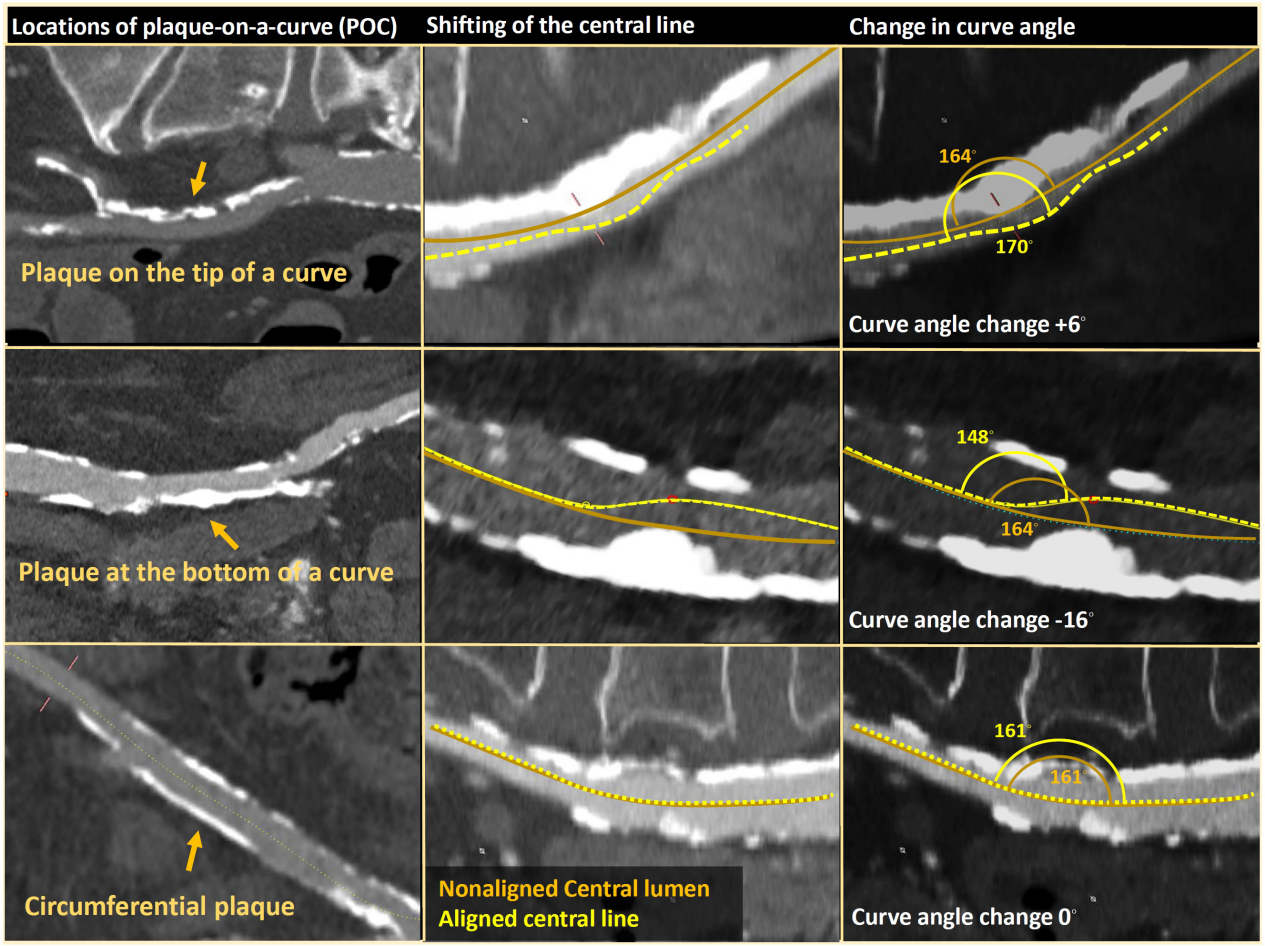
The effect of plaque-on-a-curve (POC) on curve angle.

The role of calcified plaques on crossability is known to directly affect the MLD by narrowing the free lumen. Our study identified that when the calcified plaque is positioned on a curve (POC), it affects tortuosity by changing the curve's angle by up to 10% degrees (following alignment of the central line to the plaque dimension and not the vessel's outer walls). The curvature change can be more acute or obtuse for different POC positions (Supplementary Table S3). When the plaques are in the opposite direction of the vessel curve, an incremental change in the curve angle (the angle becomes more acute) is seen. At the same time, an opposite effect (the angle becomes more obtuse) was seen when plaques were in the same direction as the vessel's curve (Supplementary Figure S4). Circumferential plaque occupying the entire vessel wall did not affect the net curve angle (Supplementary Figure S3]. Furthermore, small-diameter vessels gain a relatively higher effect on the curve's slope than large-caliber vessels (Figures 6, 7).

**Figure 8:**
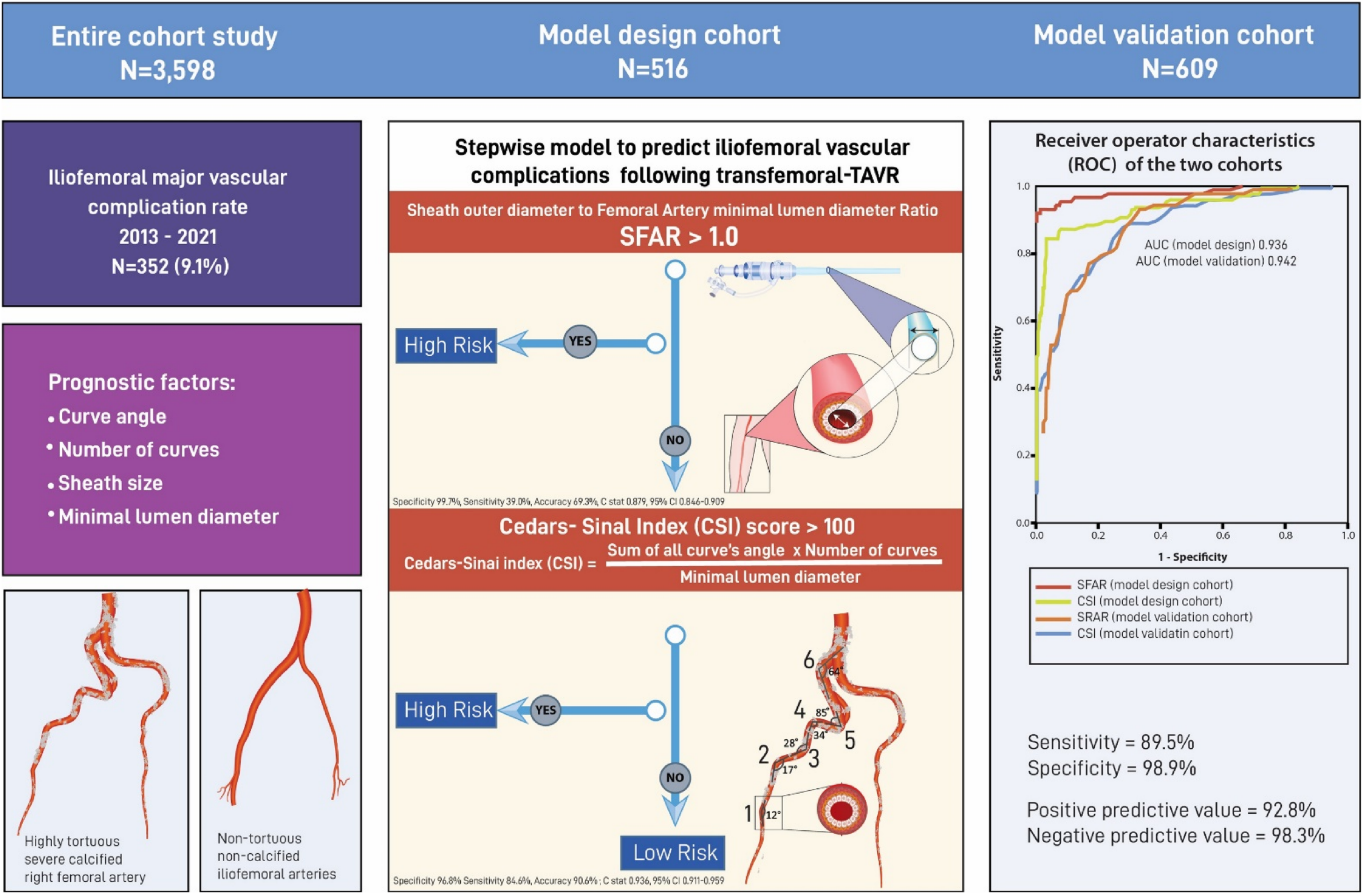
Central Illustration.

### Model validation

The validation cohort includes 609 consecutive TAVR patients enrolled in 2021. In this cohort, 42 (6.9%) patients developed IVC and 567 (93.1%) did not. The predictive model correctly identified 38 of the 42 IVC patients and labelled 4 control patients as high risk. Overall, the model predicted IVC with an 89.5% sensitivity, 98.9% specificity, and 94.2% accuracy and a positive and negative predictive value of 92.8% and 98.3%, respectively. The model showed a good discrimination ability (C-stat 0.942, 95% CI 0.904–0.980, *p* < .0001) and was well calibrated (optimism-corrected calibration slop of 0.938) [Central illustration].

## Discussion

Over the last few years, TAVR has become a widely accepted procedure in the US for symptomatic severe AS patients using the transfemoral approach. Initial experience indicated an unacceptable rate of IVC and were addressed by the manufacturers to improve valve and sheath profile.

Despite the tremendous technological improvement, IVC remain one of the most common TAVR complications (1–4), suggesting that other factors, such as the patient's vascular anatomy, play a significant role in IVC and the 2017 ACC TAVR expert consensus gathering recommended using them in routine pre-procedural planning (20).

In clinical practice, the lumen diameter is the most widely used variable to assess the minimal vascular dimension set by the manufacturer for safely crossing the iliofemoral artery. While, in most cases, the MLD, as a single variable, is enough to guide the operators in the transfemoral approach, its accuracy is much lower when the artery is tortuous and calcified (21). Over the years, numerous attempts have been made to find a way to quantify tortuosity and calcification. Hayashida et al. suggested using categorized calcification, scaled tortuosity, and the sheath-to-femoral artery ratio with a cutoff of 1.05 (22). Mach et al. used the true and ideal vessel lengths (IFT), and Hammer et al. recommended using the iliofemoral lumen volume (IFV) (23, 24).

Despite the early promising results of numerous single-center studies using sole predictor, the followed validation and the multicenter studies showed mixed and controversial results (23–25). The reason for that may be related to using a single variable for complex anatomy and requires a more profound understanding of the relation and correlation between the various factors. Moreover, previous studies include minor and major vascular complications as two distinct types based on the severity of the outcome as death, life-threatening, or major bleeding. While minor vascular complications are more common and major complications are associated with increased mortality, both affect the procedure's safety profile, alter medical care, prolong hospitalization length, and expose the patients to further procedures and potentially more complications. In our study, we decided to include the combined vascular outcome in the analysis and not to break out the two types. Our foremost concern was to provide a tool to help prevent vascular complications all together, regardless of severity. We further highlight the influence of anatomical-based variables by matching the study population and balancing significant predictors for patients' baseline characteristics and procedure-related features.

We adjusted the study's period to recent years of using the new generation transcatheter devices and used a propensity matching technique to strengthen the retrospective and observational nature of the study (26) and make it more suitable for current practice. To overcome the artery's three-dimensional complexity shape and calcified plaques' effect and the intraobserver error, we measured the iliofemoral angle using the automatic tortuosity tool and aligned the central lumen line to the plaque dimension and the free lumen. Our Cedars-Sinai score index integrates anatomical predictors into a novel and simple-to-use formula. It uses the number of curves and the sum of angles with the MLD to create a powerful discrimination tool and, in turn, allows us to understand the various reasons for IVC in complex vessels far beyond the MLD (Supplementary Figure S5).

The CSI score allows operators a quantified severity scale to optimize procedural planning. Operator responses may include balloon angioplasty with concomitant stenting, intravascular lithotripsy, surgical cutdown, intravascular US guidance, sequential upgrading of wire stiffness (i.e., Meier wire, Amplatz super stiff, Safari, Confide, Lunderquist, etc.), or referring to a tertiary center with high volume and experience. Alternatively, switch to safer and more convenient alternative access. Moreover, utilizing the CSI score and the risk model may help create a safer TAVR protocol. High-risk patients might require more frequent and diligent post-procedure checks and further reduce the risk of detecting IVC earlier.

While the primary effect of our predictive model focuses on tortuosity and MLD, we also reported that the plaque's location at the vessel modestly alters the curve angle and, potentially, for daily life practice, shifts the catheter tip. Plaque located in the same curve direction may diversify the catheter toward the opposite wall, resulting in a higher risk of vascular injury. In contrast, plaque opposite to the curve direction directs it away from the vessel wall, allowing the operator an extended range of maneuver. As much as our reports identify distinct habits, the actual effect of the POC remains unclear. It is more theoretical as plaques may vary significantly in their consistency even though no difference might be observed in CT scans.

The final predictive model, which uses our CSI score in a stepwise tree model, was validated in a new cohort using consecutive TAVR patients from 2021 and provides the highest sensitivity and accuracy of any prior model.

### Study limitation

The study is retrospective and relies on data from patient's electronic records for baseline characteristics and outcomes collected during 30-day and 1-year visits. The data was entered prospectively as part of a national registry. It was extracted for the study using data mining services that helped to eliminate human error and minimize loss of medical data from outside centers and private clinics. Furthermore, all reconstructed CT analyses for the model design and validation were performed prospectively.

The model design and validation were based on a single-center experience performing TAVR guided by TEE under general anesthesia as routine practice. This may impact the incidence of vascular complications and the overall outcome. Therefore, it should be validated by multicentered experience.

Lastly, reconstruction analysis of imaging was achieved using the 3mensio software, which may have produced reconstructions that slightly vary from those generated from alternative software, thereby requiring further verification.

## Conclusions

IVC rate has decreased over the years mainly due to technological improvement of the transcatheter device and sheath profile but remains one of the most common complications following TF-TAVR. Despite the tremendous progression, current practices use the minimal lumen diameter as a primary variable for assessing the feasibility of crossing the iliofemoral arteries. In contrast, strong predictors such as calcification and tortuosity, which are not rare in the octogenarian population of TAVR, are mainly assessed using eyeballing and rely on operator experience. The lack of a proper tool to accurately quantify tortuosity and calcification poses a higher risk for procedural complications and poor outcome.

In our study, we address the gap in knowledge by designing a score index (CSI) that aid in quantifying tortuosity and calcification from contemporarily data involving a new generation transcatheter system. The CSI is a simple-to-use formula consisting of the multiplication of the sum of the curve angles and the number of curves divided by the minimal lumen diameter. We further integrated the CSI into a two-step risk assessment model to increase its sensitivity and accuracy. The designated model was validated in a cohort compromising of consecutive patients and identified 89.5% of the IVC patients with 98.9% specificity and 94.2% accuracy, making it the most accurate tool reported.

## Data Availability

The original contributions presented in the study are included in the article/Supplementary Material, further inquiries can be directed to the corresponding author.
