# Supplementary material for: Predicting the risk of iliofemoral vascular complication in complex transfemoral-TAVR using new generation transcatheter devices
Source: Front Cardiovasc Med. 2023 Jul 6;10:1167212. doi: 10.3389/fcvm.2023.1167212 (PMC10357287; doi:10.3389/fcvm.2023.1167212)
Supplement: Supplementary file 1 [file Datasheet1.docx]

**Predicting the Risk of Vascular Complications in Transfemoral-TAVR using the New Generation Devices**

Ofir Koren, MD^a,b^, Vivek Patel, MS,^a^ Yuval Tamir, MS^c^, Keita Koseki, MD,^a,d^, Danon Kaewkes, MD,^e^ Troy Sanders, MS^f^, Robert Naami^f^, MD, Edmund Naami, BS^h^, Daniel Eugene Cheng, BA^a^ , Sharon Shalom Natanzon, MD^a^, Alon Shechter, MD, MHA^aij^, [Jeffrey Gornbein](https://pubmed.ncbi.nlm.nih.gov/?term=Gornbein+J&cauthor_id=31515123), DrPH^f^, Tarun Chakravarty, MD,^a^ Mamoo Nakamura, MD,^a^ Wen Cheng, MD,^a^ [Hasan Jilaihawi](https://pubmed.ncbi.nlm.nih.gov/?term=Jilaihawi+H&cauthor_id=26493666), MD ^a^, Raj R. Makkar, MD^a^

^a^ Cedars-Sinai Medical Center, Smidt Heart Institute, Los Angeles, California

^b^ Bruce Rappaport Faculty of Medicine, Technion Israel Institute of Technology, Haifa, Israel

^c^ Weizmann Institute of Science, Rehovot, Israel

^d^ Department of Cardiology, The University of Tokyo Hospital, Tokyo, Japan

^e^ Queen Sirikit Heart Center of the Northeast, Department of Medicine, Faculty of Medicine,

Khon Kaen University, Khon Kaen, Thailand

^f^ David Geffen School of Medicine, University of California (UCLA), Los Angeles, California

^g^ Internal Medicine, University Hospitals Cleveland Medical Center, Case Western Reserve

University School of Medicine, Cleveland

^h^ School of Medicine, University of Illinois, Illinois

^i^ Faculty of Medicine, Tel Aviv University, Tel Aviv, Israel

^J^ Department of Cardiology, Rabin Medical Center, Petach Tikva, Israel

^k^ Heart Valve Center, NYU Langone Health, New York.

**Running title: Predictive Model for vascular complication in TAVR**

**Conflict of interest:** None

**Author disclosures:** Dr. Makkar received grant support from Edwards Lifesciences Corporation; he is a consultant for Abbott Vascular, Cordis, and Medtronic and holds equity in Entourage Medical. Dr. Chakravarty is a consultant, proctor, and speaker for Edwards Lifesciences and Medtronic; he is a consultant for Abbott Lifesciences and a consultant and speaker for Boston Scientific. Other authors have no conflicts of interest to disclose.

**Address for correspondence:** Raj R. Makkar, MD

Cedars-Sinai Smidt Heart Institute

127 S. San Vicente Boulevard, Advanced Health Sciences Pavilion

Third Floor, Suite A3100, Los Angeles, CA 90048

E-mail: Raj.Makkar@cshs.org

Tel: 310-423-3277; Fax: 310-423-0166

**Supplementary**

**Tables**

**Table 1s.** Patients’ characteristics, procedural data, and outcome among unmatched study group

**Table 2s.** logistic regression model using the SFAR and the CSI score for the prediction of iliofemoral vascular complications

**Table 3s.** The effect of plaque location on curve angle

**Table 1s.** Patients’ characteristics, procedural data, and outcome among unmatched study group

|  | Total Cohort  N=2989 | IVC Group  N=266 | Control Group  N=2723 | *p-Value* | |
| --- | --- | --- | --- | --- | --- |
|  |  |  |  | *Unadjusted* | *Adjusted for Sex and PAD* |
| Age (years), median (±IQR) | 81.1 (12) | 82.7 (10) | 80.9 (12) | <.0001 |  |
| Female Sex, n (%) | 1173 (39.2) | 131 (49.2) | 1042 (38.3) | .001 | .008 |
| Smoking, n (%) | 128 (4.3) | 5 (1.9) | 123 (4.5) | .039 | .215 |
| BMI, n (%) | 26.6 (6.7) | 25.8 (7.0) | 26.6 (7.0) | .070 |  |
| CKD ≥ III, n (%) | 502 (16.8) | 53 (19.9) | 449 (16.5) | .169 |  |
| Dialysis, n (%) | 152 (5.1) | 13 (4.9) | 139 (5.1) | 1.000 |  |
| COPD, n (%) | 481 (16.1) | 54 (20.3) | 427 (15.7) | .055 | .540 |
| Hyperlipidemia, n (%) | 1598 (53.5) | 143 (53.8) | 1455 (53.4) | .949 |  |
| Diabetes Mellitus, n (%) | 952 (31.9) | 77 (29.1) | 875 (32.2) | .334 |  |
| Peripheral artery disease (PAD), n (%) | 427 (14.3) | 63 (23.7) | 364 (13.4) | <.0001 |  |
| Coronary artery disease, n (%) | 1284 (43.0) | 119 (44.7) | 1165 (42.8) | .559 |  |
| CVA/TIA, n (%) | 359 (12.0) | 36 (13.5) | 323 (11.9) | .429 |  |
| Myocardial Infarction, n (%) | 349 (11.7) | 28 (10.5) | 321 (11.8) | .617 |  |
| CABG surgery, n (%) | 436 (14.6) | 45 (16.9) | 391 (14.4) | .275 |  |
| Porcelain Aorta, n (%) | 102 (3.4) | 8 (3.0) | 94 (3.5) | .860 |  |
| STS risk score, median (±IQR) | 3.6 (3.7) | 5.0 (4.3) | 3.5 (3.6) | <.0001 | .057 |
| NYHA Class ≥ III, n (%) | 2363 (79.1) | 220 (82.7) | 2413 (78.8) | .134 |  |
| Five minutes’ walk test (m), median (±IQR) | 15.3 (5.7) | 15.3 (4.3) | 15.3 (5.7) | .357 |  |
| KCCQ Score, median (±IQR) | 53.2 (38.6) | 51.3 (37.7) | 56.2 (39.1) | .043 | .124 |
| Balloon-expandable THV, n (%) | 2442 (81.7) | 205 (77.1) | 2237 (82.2) | .046 | .050 |
| Old Generation THV, n (%) | 415 (13.9) | 55 (20.7) | 360 (13.2) | .001 | .067 |
| Sheath size ≥ 16 F, n (%) | 596 (19.9) | 75 (28.2) | 521 (19.2) | .001 | .002 |
| Aortic valve area (mm), median (±IQR) | 0.7 (0.2) | 0.7 (0.3) | 0.7 (0.2) | .846 |  |
| Calcium score – Coronaries (HU), median (±IQR) | 989 (1886) | 923 (1692) | 995 (1895) | .882 |  |
| Calcium score – Aortic (HU), median (±IQR) | 2527 (2143) | 2341 (2442) | 2556 (2106) | .179 |  |
| LVEF (%)^,^ median (±IQR) | 64 (12) | 64 (12) | 64 (12) | .769 |  |
| **BMI, Body mass index; CKD,** Chronic kidney disease stage ≥ III**; COPD**, Chronic obstructive lung disease; **CVA/TIA**, Cerebrovascular accident/Transient ischemic attack; **CABG**, coronary artery bypass graft; **KCCQ**, Kansas City Cardiomyopathy Questionnaire; **THV**, Transcatheter heart valve; **HU**, Hounsfield units; **LVEF**, left ventricular ejection fraction; **PACU**, post anesthesia care unit; **RBC**, red blood cells.  ^¥^Large THV referred to 29 mm Sapien valve or 34 mm Evolute valve | | | | | |

**Table 2s.** logistic regression model using the SFAR and the CSI score for the prediction of iliofemoral vascular complications

| **Predictors** | **B** | **SE(B)** | ***p*-value** |
| --- | --- | --- | --- |
| Intercept | -30.19 | 3.14 | <.0001 |
| SFAR > 1.00 | 10.98 | 1.27 | <.0001 |
| log CSI ^µ^ > 100 | 11.00 | 1.43 | <.0001 |
|  | C-stat (ROC) | Nagelkerke R square | logit threshold |
|  | 0.976 | 0.820 | -0.742 |
| **SFAR**, sheath outer diameter to femoral artery MLD ratio; **CSI**, Cedars-Sinai index.  ^µ^ CSI index = (Sum of all angles times the number of curves) divided by the lowest minimal lumen diameter  log CSI is log base 10 of CSI  Using both predictor: Specificity 91.9%; Sensitivity 92.4%; Accuracy 92.9% | | | |

**Table 3s.** The effect of plaque location on curve angle

|  | *Position of plaque* | *Lumen diameter* | *Curve’s angle Centralized to vessels wall* | *Curve angle Centralized to plaque wall* | *Degree of angle change* |
| --- | --- | --- | --- | --- | --- |
| 1 | Tip of the vessel | 5.7 | 45 | 52 | ↑ 7 (15.5) |
| 2 | Tip of the vessel | 6.2 | 62 | 66 | ↑ 4 (6.4) |
| 3 | Tip of the vessel | 5.9 | 48 | 54 | ↑ 6 (12.5) |
| 4 | Tip of the vessel | 7.4 | 34 | 39 | ↑ 5 (14.7) |
| 5 | Tip of the vessel | 8.2 | 26 | 28 | ↑ 2 (4.6) |
| 6 | Base of the vessel | 5.5 | 32 | 28 | ↓ 4 (12.5) |
| 7 | Base of the vessel | 6.4 | 44 | 41 | ↓ 3 (6.8) |
| 8 | Base of the vessel | 6.8 | 55 | 50 | ↓ 5 (9.1) |
| 9 | Base of the vessel | 7.4 | 26 | 22 | ↓ 4 (15.3) |
| 10 | Base of the vessel | 9.4 | 37 | 34 | ↓ 3 (8.1) |
| 11 | Circumflex | 5.8 | 42 | 42 | 0 (0) |
| 12 | Circumflex | 5.5 | 45 | 45 | 0 (0) |
| 13 | Circumflex | 6.9 | 36 | 36 | 0 (0) |
| 14 | Circumflex | 6.4 | 49 | 49 | 0 (0) |
| 15 | Circumflex | 8.4 | 68 | 68 | 0 (0) |

**Figures legends**

**Figure 1s.** Step by step guide for curve’s angle assessment in calcified and tortuous vessel

**Figure 2s.** Standard deviation before (A) and after (B) propensity matching for entire matched population and for each variable (C)

**Figure 3s.** Correlation of significant anatomical predictors and IVC probablity

**Figure 4s.** The effect of POC and curve-direction on IVC risk

**Figure 5s.** CSI score of patients with and without IVC of different MLD and tortuosity

**
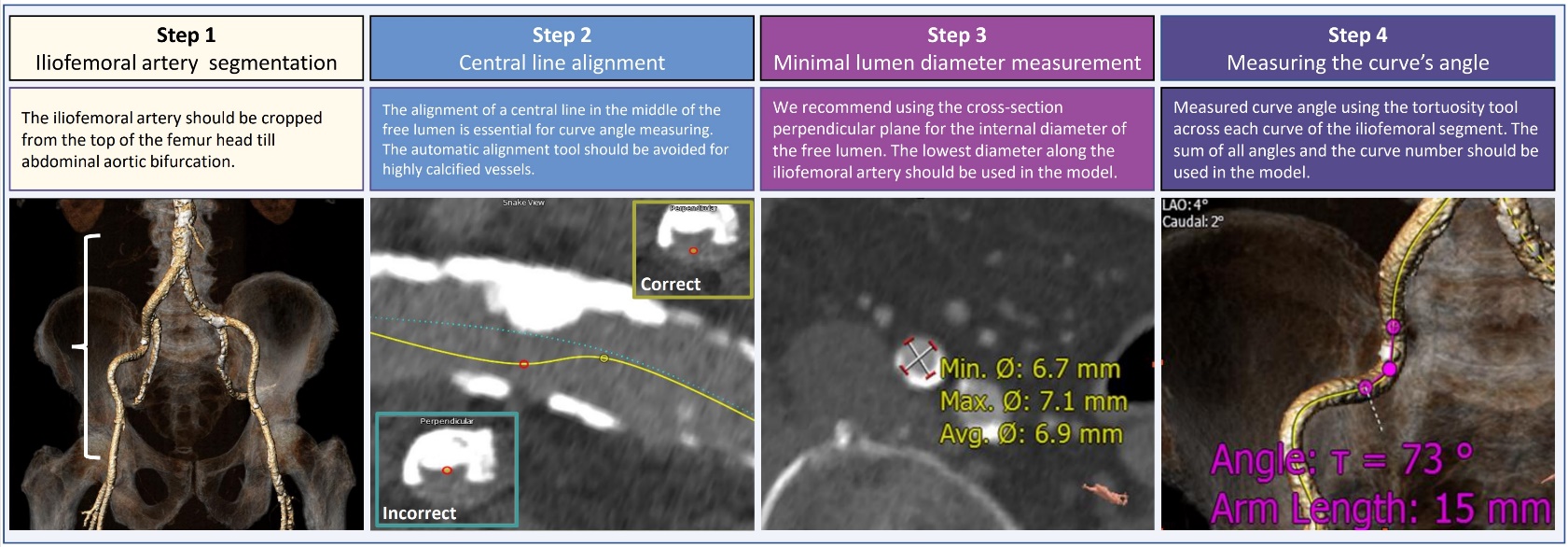
Figure 1s.** Step by step guide for curve’s angle assessment in calcified and tortuous vessel

**Figure 2s.** Standard deviation before (A) and after (B) propensity matching for entire matched population and for each variable (C)

**
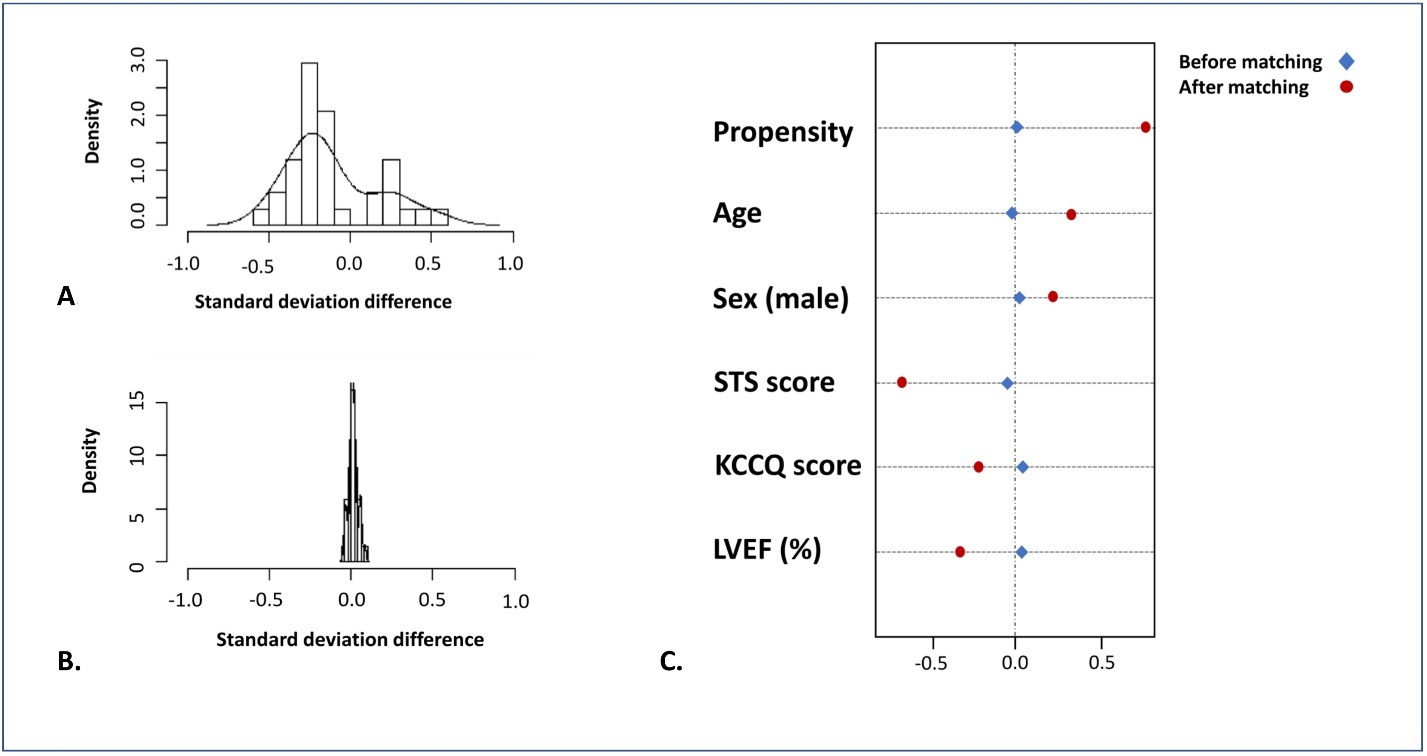
**

**
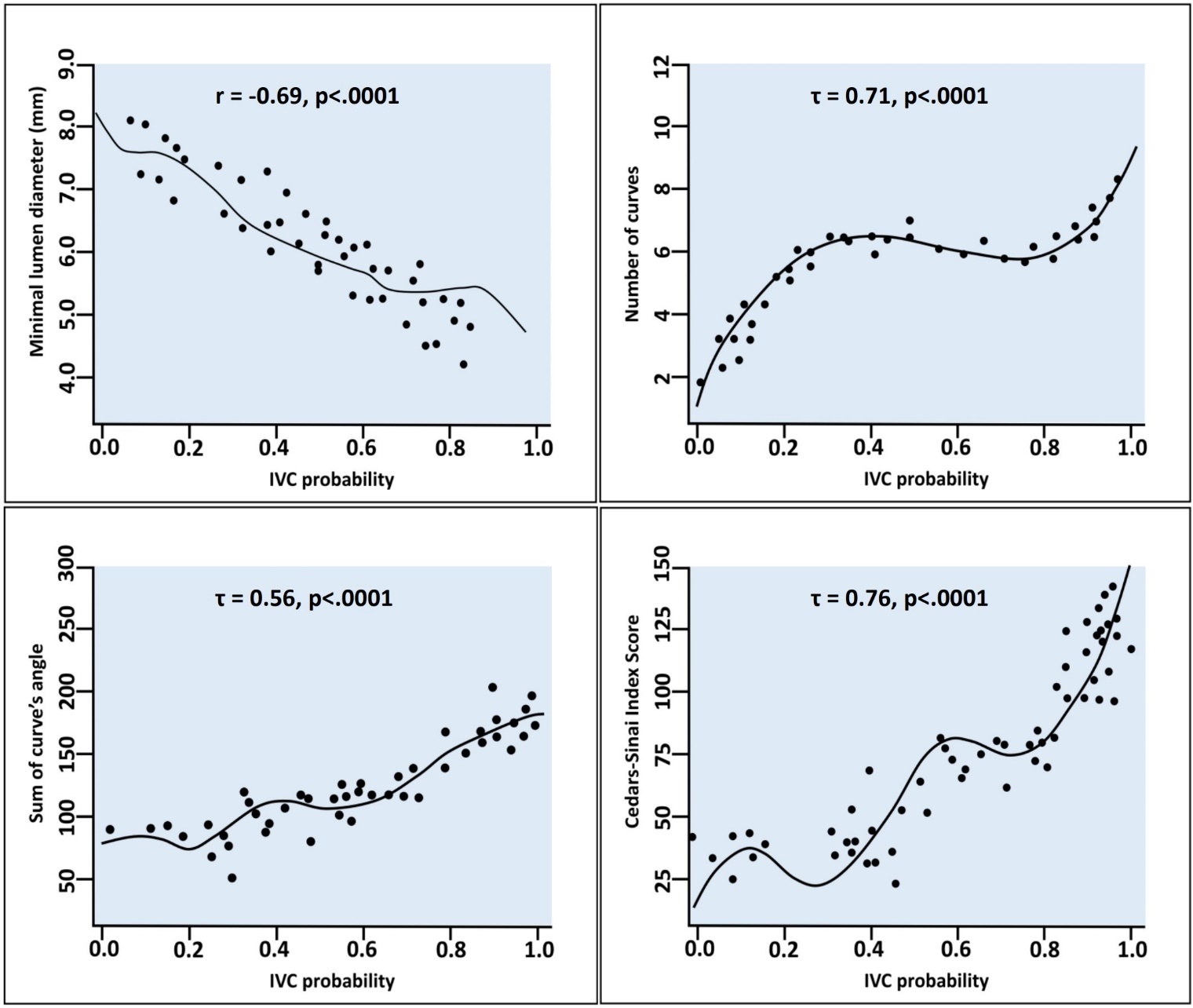
Figure 3s.** Correlation of significant anatomical predictors and IVC probablity

**
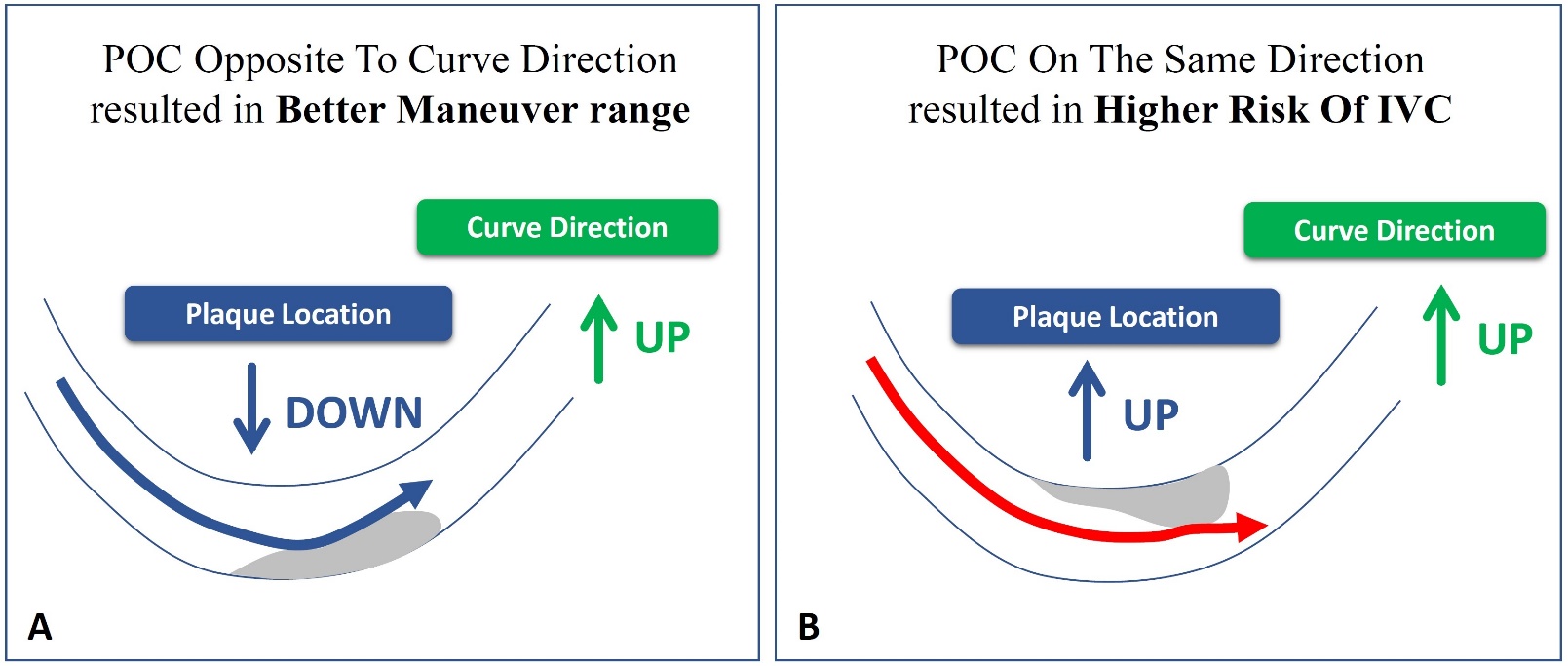
Figure 4s.** The effect of POC and curve-direction on IVC risk

**
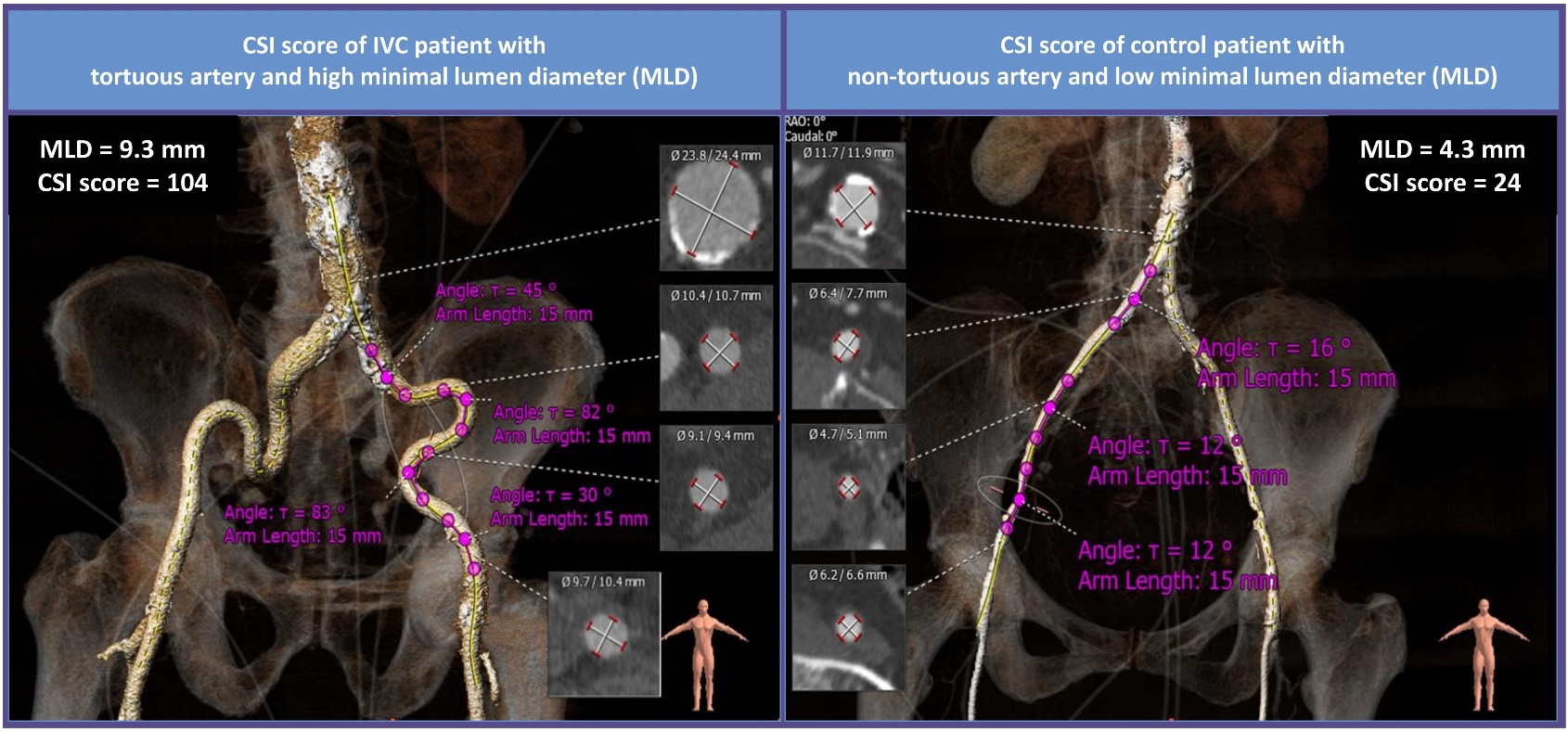
Figure 5s.** CSI score of patients with and without IVC of different MLD and tortuosity
